# Adipose Tissue Dynamics, Thermogenesis, and Interorgan Connections for Preventing Obesity and Metabolic Disorders

**DOI:** 10.31662/jmaj.2023-0218

**Published:** 2024-03-18

**Authors:** Masaji Sakaguchi

**Affiliations:** 1 Department of Metabolic Medicine, Faculty of Life Sciences, Kumamoto University, Kumamoto, Japan

**Keywords:** adipokines, adipose tissue remodeling, brown adipocyte, insulin resistance, interorgan connections, thermogenesis, type 2 diabetes, UCP1

## Abstract

Adipose tissues, such as white, brown, and beige tissues, play pivotal roles in maintaining energy balance and metabolic health. Whereas white adipocytes store energy, brown and beige adipocytes exhibit high energy expenditure owing to their distinct mitochondrial density and UCP1 expression. Dysfunction in these tissues contributes to metabolic disorders such as type 2 diabetes and cardiovascular diseases. Adipose tissue expansion through cell enlargement or increased cell numbers caused by excess energy storage in white adipocytes substantially influences metabolic health. In obesity, hypertrophic adipocytes trigger inflammation, fibrosis, and hypoxia, whereas smaller adipocytes exert favorable metabolic effects, contributing to insulin sensitivity.

Brown and beige adipocytes consume energy for thermogenesis to maintain body temperature, contributing to metabolic homeostasis. The intricate interactions between brown adipose tissues and various organs, such as the liver and heart, highlight the systemic implications of adipose tissue functions. Understanding the complex underlying mechanisms may lead to the development of innovative therapies targeting metabolic disorders by modulating the functions of brown adipose tissue and its interactions with other physiological systems. In this review, we discuss insights into the mechanisms underlying the dysregulation of metabolism owing to abnormalities in adipose tissue remodeling. We focus on the endocrine functions of thermogenic brown and beige adipocytes and explore the interorgan interactions that influence whole-body metabolism.

## Introduction

Obesity caused by insufficient exercise and overeating leads to increased visceral fat tissue mass, resulting in insulin resistance, and the development of metabolic diseases such as type 2 diabetes. Various pathological alterations trigger the progression of obesity. According to a model by Mehran AE, excessive intake of carbohydrates induces energy accumulation, leading to increased glucose uptake in fat tissues through insulin action. This process enlarges adipocytes and increases lipid content, suggesting a potential role of insulin in driving obesity ^(1)^. However, contradictory findings indicate that individuals with metabolic diseases often exhibit insulin resistance, which is correlated with higher fasting insulin levels in the blood ^(1), (2), (3)^.

Despite the contradictory findings, obesity is considered to be primarily caused by energy intake and expenditure imbalance. Atwater et al. considered energy to be a unit of calories, and proposed that it could be consumed by being stored, burned, or emitted ^(4)^. In obesity, the body accumulates more energy than it consumes ^(5)^; subsequently, dietary restrictions have become the cornerstone of obesity treatment.

Recent studies of adipose tissue have reported its diverse functions beyond energy storage, including energy expenditure, adipokine and cytokine secretion, and inflammatory responses to tissue damage ^(6)^. Adipose tissues undergo various alterations in response to environmental changes, heat and cold weather, nutritional conditions, and individual locomotor movements ^(7)^. Notably, two types of adipose tissue play pivotal roles in maintaining metabolic balance, namely, white adipose tissue (WAT), which is used for energy storage, and brown adipose tissue (BAT), which is used for energy expenditure and heat production ^(8)^.

## White and Brown Adipocytes

**Figure 1.**
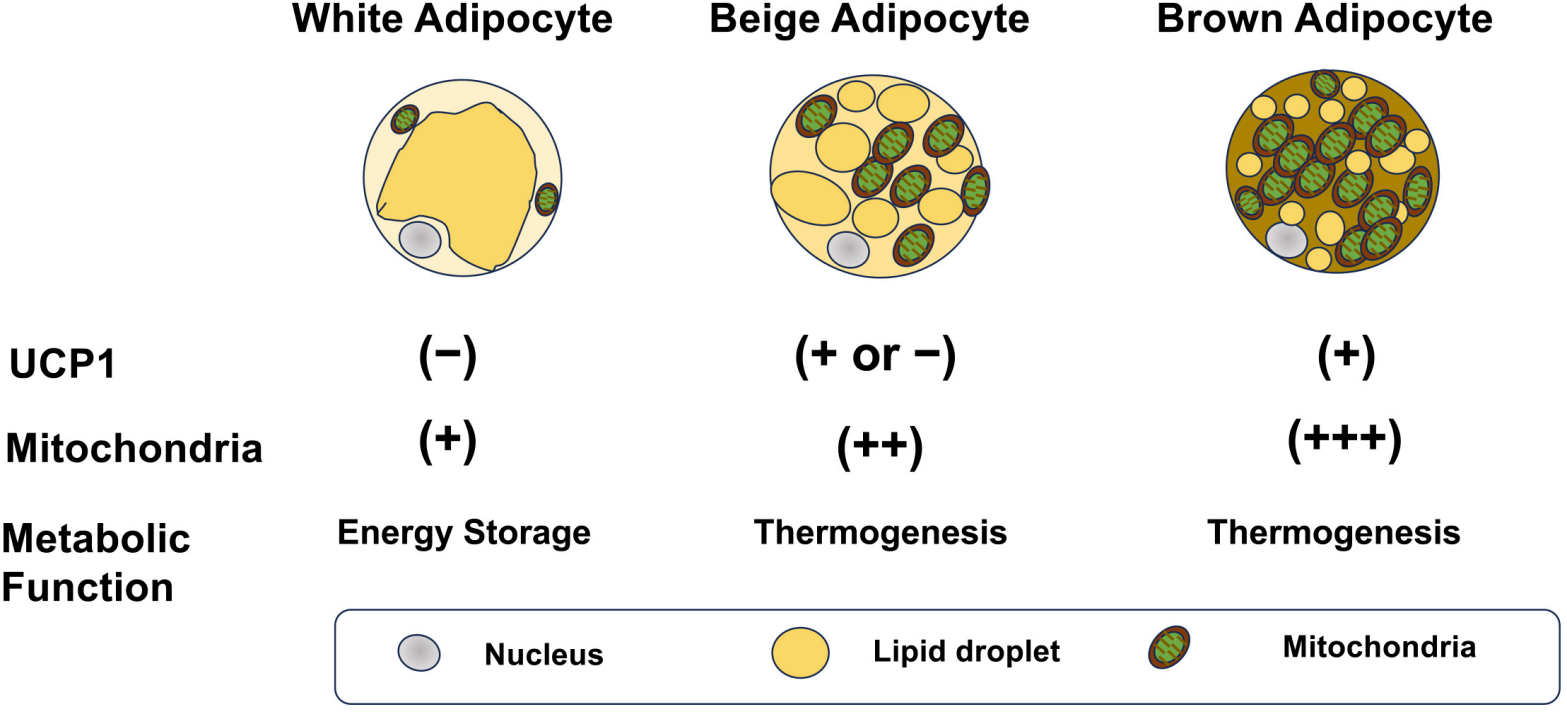
Adipocytes and their functions.

Adipose tissues are mainly classified into WAT and BAT, which exhibit substantially distinct functions and morphologies. White adipocytes typically contain large unilocular lipid droplets and relatively fewer mitochondria, and represent the classic perception of adipose tissue as an energy storage site ^(9)^. Conversely, brown adipocytes have several small multilocular lipid droplets and a high mitochondrial density and exhibit an enhanced capacity for energy expenditure ^(10)^. In addition, a subset of white adipocytes, known as beige adipocytes, have characteristics similar to those of brown adipocytes in response to stimuli such as cold exposure, exercise training, and pharmacological activation of β-adrenergic receptors (Figure 1). Fat thermogenesis mainly stems from the high mitochondrial density and expression of uncoupling protein 1 (UCP1) in brown and beige adipocytes. UCP1 is located at the inner mitochondrial membrane and dissipates energy as heat by transporting protons back from the mitochondrial intermembrane space to the matrix without generating ATP ^(7), (8)^. This process uncouples glucose and fatty acid metabolism from ATP production, releasing energy as heat. Furthermore, brown and beige fat tissues substantially regulate systemic metabolism by releasing various secretory signaling molecules (proteins, cytokines, lipids, microRNAs [miRNAs], etc.) into the circulation ^(11), (12)^.

In humans, UCP1-positive adipose tissues have been confirmed to exist in several depots, including the neck, supraclavicular region, perirenal area, and paravertebral regions. The negative correlation between body mass index (BMI) and BAT activity in humans indicates the potential of BAT activation as a promising target for obesity treatment. Moreover, studies in humans and mice have demonstrated a positive correlation between the level of activated BAT and insulin sensitivity, hinting at the potential application of strategies to augment BAT quantity or activity in obesity and diabetes treatment ^(13)^.

## Adipose Tissue Remodeling and Inflammatory Crosstalk

The expansion of adipose tissue is closely associated with metabolic health. In general, a high fat mass corresponds to poor metabolic health. However, the high capacity for adipose tissue expansion exerts a protective effect against metabolic disorders. This seemingly contradictory phenomenon can be explained by considering how excess nutrients are processed. Excess absorbed nutrients need to either be used as energy or stored as lipids. WAT is the only tissue capable of safely storing large amounts of excess nutrients, such as lipids. Therefore, efficient storage of excess nutrients in WAT and their uptake into thermogenic fat for heat generation support metabolic health ^(14)^.

Notably, the location and manner in which adipose tissue expands, whether through hyperplasia (increased cell number) or hypertrophy (increased cell size), substantially impact metabolic health. Adipocyte hypertrophy and hyperplasia strongly contribute to adipose tissue expansion. Hypertrophic growth involves large adipocytes, whereas hyperplasia involves an increase in the number of adipocytes. Hyperplasia and hypertrophy of adipocytes can lead to inflammation, fibrosis, and hypoxia in adipose tissue, potentially worsening metabolic health ^(15)^. Adipocytes can rapidly expand or contract in response to dietary changes. This dynamic process is driven by changes in stromal vascular cells, preadipocyte cells, and immune cells. These changes collectively define “adipose tissue remodeling,” enabling adaptation to various metabolic states ^(16)^. Adipocyte hyperplasia and hypertrophy can lead to various effects, including hypoxia, inflammation, fibrosis, adipocyte cell death, excessive chemical secretion, and disruption of the fatty acid balance. These effects are associated with adipocytes and require macrophages to create a suitable environment for the remodeling process. As a result, macrophages form crown-like structures surrounding necrotic adipocytes in advanced obesity. These cells phagocytose lipid remnants, forming large lipid-containing multinucleated cytoplasm, which is a sign of chronic inflammation ^(17)^.

The pathologic remodeling of adipose tissue and insulin resistance affects broader adipose remodeling that involves extracellular matrix changes ^(16)^. These changes are associated with chronic low-grade inflammation during overnutrition and contribute to the development of insulin resistance in individuals with type 2 diabetes. Adipocyte hypertrophy leads to localized microhypoxia within the adipose tissue during early expansion ^(18)^. Recent clinical observations indicate an insufficient oxygen supply in obesity ^(19)^. Studies that used mouse adipocytes and animal models have demonstrated that hypoxia increases the levels of inflammatory adipokines, including macrophage migration inhibitory factor, matrix metalloproteinase (MMP)2 and MMP9, PAI-1, VEGF, Angplt4, IL1 and IL-6, and TNF-α ^(17)^. Enlargement of the adipose tissue due to hypertrophy is a risk factor for metabolic syndrome ^(20)^, independent of BMI. Interestingly, nonobese individuals with insulin resistance and diabetes often have large hypertrophic adipocytes, which is associated with metabolic dysfunction rather than overall adipose tissue mass ^(21)^. Larger adipocytes increase lipolysis rates and the levels of inflammatory cytokines, whereas smaller adipocytes may increase the secretion of adiponectin, a hormone that improves insulin sensitivity ^(22)^.

## Thermogenesis in the Adipose Tissue

Prolonged exposure to cold temperatures increases BAT mass and the expression of thermogenic genes, resulting in greater thermogenic capacity ^(23)^. In rodents, cold exposure triggers the development of mitochondria-rich, thermogenic beige adipocytes within the WAT, a process known as “browning” or “beiging.” Beige adipocytes presumably originate through multiple mechanisms: new beige adipocyte formation from progenitor cells, conversion of mature adipocytes into beige adipocytes, and multiplication of mature adipocytes. The primary physiological signal that controls brown and beige adipocyte formation and thermogenic activity is adrenergic signaling through the sympathetic nervous system ^(24)^. Adipose tissue, particularly BAT, is connected to a dense network of sympathetic neurons. Exposure to cold temperature prompts the release of norepinephrine (NE) from sympathetic neurons, activating the β-adrenergic receptor. It triggers the activation of adenylate cyclase to produce cyclic AMP, leading to the subsequent activation of protein kinase A (PKA), which phosphorylates and activates hormone-sensitive lipase and the transcription factor CREB ^(25)^. This cascade triggers lipolysis and thermogenesis facilitated by PPARγ coactivator-1a (PGC1-a), and uncoupling of the electron transport chain through the action of UCP1. When phosphorylated by p38 mitogen-activated protein kinase (MAPK), PGC1-a is activated in response to β-adrenergic signaling ^(26)^. Adrenergic stimulation of adipocytes also activates the mTOR pathway, a core regulator of cell and tissue metabolism.

Moreover, several studies have reported other mechanisms through which adipocytes can induce thermogenesis independently of β3-adrenergic ligands. Some of these mechanisms involve signaling by other various external signals, hormones, and metabolites (such as natriuretic peptides and acetylcholine) ^(7)^. In addition to the classic β3-adrenergic signaling pathway, other mechanisms have been shown to independently induce thermogenic machinery in the adipose tissue. This expanded perspective highlights the complexity of thermoregulation in organisms.

## Crosstalk of BAT and Other Organs

Fibroblast growth factor 21 (FGF21) is an endocrine regulatory factor that is mainly produced in the liver and adipose tissues; it engages in direct interactions between adipose tissues, particularly BAT, and the liver ^(27), (28)^. FGF21 is known to exert preventive effects against hepatic steatosis and steatohepatitis. The fundamental mechanisms through which FGF21 ameliorates hepatic fatty diseases involve decreasing lipogenesis, inducing fatty acid β-oxidation, enhancing hepatic insulin sensitivity, and reducing the delivery of very-low-density lipoprotein to the liver ^(29), (30)^. Furthermore, FGF21 has been shown to induce insulin sensitivity and augment glucose uptake in WAT ^(30), (31)^. Several studies have reported that FGF21 promotes WAT beiging, activates BAT, increases energy expenditure, sustains body temperature during cold exposure, and favors body weight loss ^(30), (32)^.

MicroRNAs secreted from adipose tissues have also emerged as mediators of signal transduction to the liver. miRNAs contained within extracellular vesicles derived from BAT regulate the expression and secretion of FGF21 in the liver ^(33), (34)^. In ADicerKO mice lacking adipose-specific miRNA processing, BAT transplantation restored circulating miRNA levels and improved glucose tolerance. This study demonstrated an increase in miR-99b after BAT transplantation, which explicitly regulates FGF21 production in the liver ^(34)^. There is also evidence of a correlation between FGF21 secretion from BAT and cardiovascular function. FGF21 released from BAT through the activation of adenosine receptor (A2aR) signaling has been shown to protect against hypertensive cardiac remodeling in a hypertensive rat model ^(35), (36)^. The inhibition of FGF21 secretion from BAT hindered recovery from heart injuries, indicating that the interaction between BAT and the heart is mediated by FGF ^(26), (36)^. Recent human studies have demonstrated that the presence of BAT suppresses the onset of cardiovascular metabolic diseases. A study examining more than 50,000 18F-FDG PET/CT reports showed that the presence of BAT reduces the risk of developing dyslipidemia, coronary artery disease, cerebrovascular disease, congestive heart failure, and hypertension ^(37)^.

Exercise has been reported to induce adaptations in BAT and WAT, including the activation of brown fat and the browning of white fat. These adaptations presumably resulted from changes in energy demands and alterations in the secretion of muscle-derived factors (myokines) that affect the function of adipose tissues. In addition, brown adipocyte-derived factors, namely, batokines, induced by cold exposure or exercise have been reported. For instance, 12, 13-diHOME has been identified as a cytokine induced by cold or exercise in mice and humans ^(38), (39)^. It reportedly enhances fatty acid utilization in brown fat and promotes fatty acid uptake and oxidation in myocytes ^(39)^. Furthermore, the underlying mechanism of the interaction between BAT and muscle mediated by the secretion of myostatin (GDF8) from BAT has been elucidated. Loss of the transcription factor IRF4 in brown fat in mice increased myostatin levels, reduced exercise capacity ^(40)^, led to abnormalities in white vastus muscles, and impaired mitochondrial function in myocytes.

**Figure 2.**
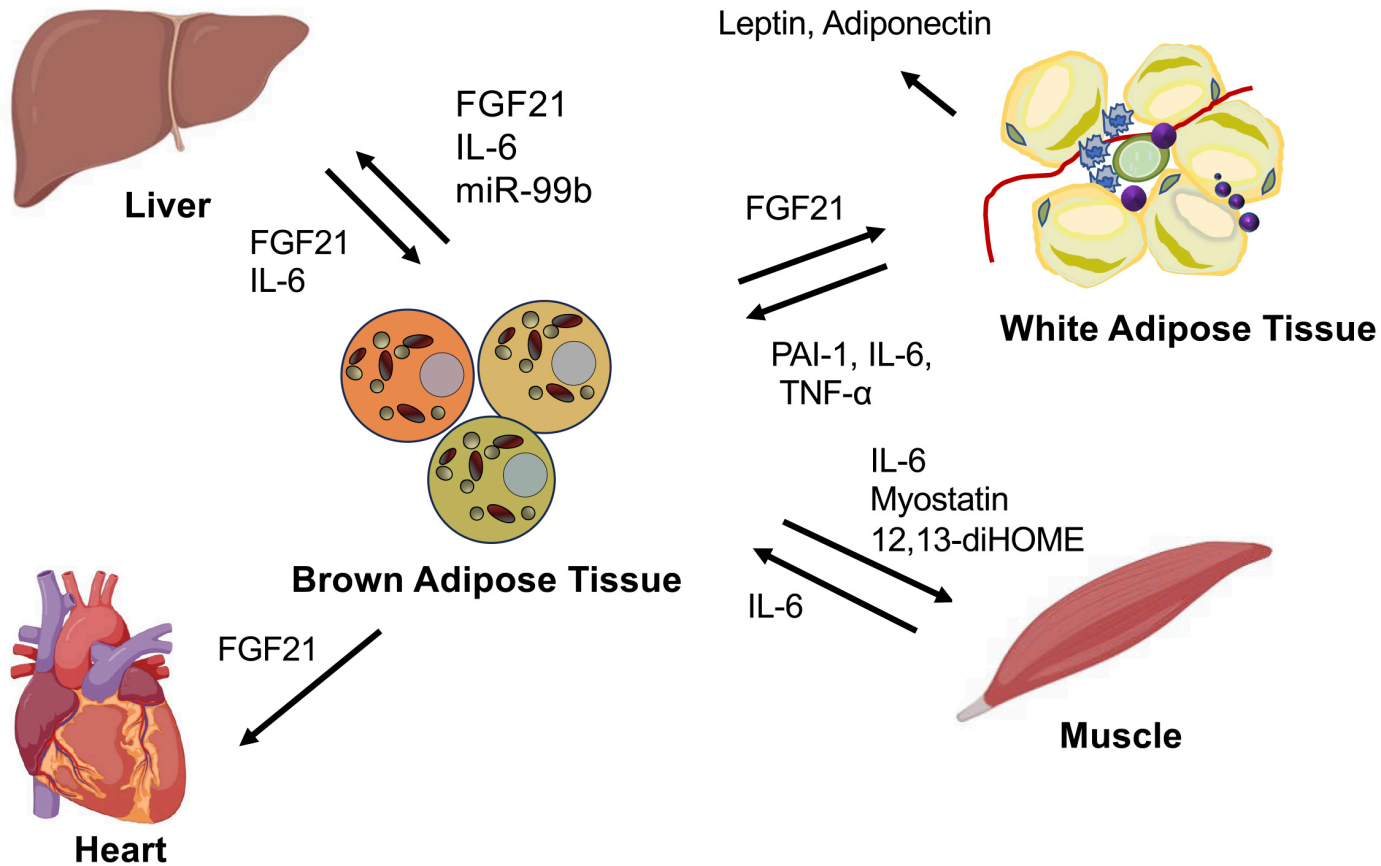
Crosstalk of brown adipose tissue with other organs. Figure 2 is generated with BioRender (https://www.biorender.com).

An increase in myostatin expression in BAT and in myostatin circulation was reported when mice were housed at thermoneutrality compared with room temperature, which led to decreased exercise capacity. IL-6, a myokine secreted during exercise, is necessary for the activation of brown fat and browning of white fat according to studies using IL-6-deficient mouse models ^(41), (42)^. Exercise-induced myokines, such as irisin, lactate, and β-aminoisobutyric acid, have also been reported to promote the activation of brown and beige fat tissues ^(43), (44), (45)^. These findings indicate that mutual communication between brown fat and muscle is necessary for maintaining the energy balance between utilization and storage in response to physiological demands (Figure 2).

## Conclusion

The dynamic interplay between adipose tissue remodeling and thermogenesis in BAT and between the systemic interactions of adipose tissues and various organs indicates the intricate mechanisms governing metabolic health. Sufficient understanding of these intricate mechanisms could lead to promising avenues for developing innovative therapeutic approaches aimed at combating obesity, type 2 diabetes mellitus, and related metabolic disorders. Pharmacological or technological interventions are expected to establish a physiological approach by stimulating BAT activation, thus augmenting energy expenditure and alleviating obesity.

## Article Information

This article is based on the study, which received the Medical Research Encouragement Prize of The Japan Medical Association in 2022.

### Conflicts of Interest

None

### Sources of Funding

This work was supported by JSPS KAKENHI (JP 21K08532).

### Author Contributions

M.S. wrote the manuscript.
